# Pesticide Use and Safety Behaviors Among Farmers in Rwanda’s Eastern Province: Perspectives from Sector Officers on Drivers, Factors, and Gender Differences

**DOI:** 10.3390/ijerph23060771

**Published:** 2026-06-08

**Authors:** Emmanuel Irimaso, Concorde Rwibutso, Therese Nyirahabimana, Cynthia Curl, Stephanie Mitchell, Liberata Mukamana, Dawn Zimmerman, Sabrina B. Sholts

**Affiliations:** 1 School of Veterinary Medicine, University of Rwanda, Nyagatare P.O. Box 57, Rwanda; irimasoemmy@gmail.com; 2 Rwanda Biomedical Centre, Kigali P.O. Box 7162, Rwanda; rizereconcorde@gmail.com; 3 Rwanda Biosolution, Kigali, Rwanda; ntherese22@gmail.com; 4 School of Public and Population Health, Boise State University, Boise, ID 83706, USA; cynthiacurl@boisestate.edu; 5 Center for One Health Research, University of Washington, Seattle, WA 98195, USA; smitch88@uw.edu; 6 School of Economics, University of Rwanda, Kigali P.O. Box 4285, Rwanda; liberatamukamana@gmail.com; 7 Department of Epidemiology of Microbial Disease, Yale School of Public Health, New Haven, CT 06510, USA; zimmermand@si.edu; 8 Department of Entomology, National Museum of Natural History, Smithsonian Institution, Washington, DC 20560, USA; 9 Department of Anthropology, National Museum of Natural History, Smithsonian Institution, Washington, DC 20560, USA

**Keywords:** pesticides, one health, agriculture, health risks, Rwanda

## Abstract

**Highlights:**

**Public health relevance—How does this work relate to a public health issue?**

- Pesticide misuse by farmers increases their risks for acute and long-term health effects, including certain cancers, respiratory impairment, and reproductive problems.
- Pesticide exposure affects farmers, livestock, wildlife, and ecosystems, creating interconnected risks for human, animal, and ecological health.

**Public health significance—Why is this work of significance to public health?**

- This study links farmers’ pesticide exposure in Rwanda to broader global challenges, such as climate change and pest resistance.
- This work is one of the first detailed, locally informed examinations of pesticide-related health risks in Rwanda based on insights from agricultural and veterinary extension officers.

**Public health implications—What are the key implications or messages for practitioners, policy makers and/or researchers in public health?**

- There is an urgent need for more information and awareness about pesticide-related health risks among farmers and extension professionals, especially in climate-stressed regions.
- Extension officers serve as key intermediaries between public health researchers and farmers in sub-Saharan Africa, offering critical insights for informing community engagement and risk communication.

**Abstract:**

Growing pesticide use is linked to increased health risks for farmers across Africa, due to factors such as improper handling, insufficient knowledge, and lack of resources. To better understand these risk factors in Rwanda, where the majority of the population and most women are farmers, we held focus group discussions (FGDs) with 31 Sector Agricultural Officers (SAOs) and Sector Animal Resources Officers (SAROs) in five districts of Eastern Province. Among their views on this topic, we identified four core themes among the FGDs of (1) interconnected challenges, (2) shared exposure and health risks, (3) gender differences in risk behavior, and (4) transmission of knowledge, in addition to six pesticides—abamectin, cypermethrin, lambda-cyhalothrin, profenofos, mancozeb, and metalaxyl—most commonly used across all districts. Our findings suggest that Rwandan farmers may overestimate variety in the pesticides that they use and thus potentially contribute to problems such as pest resistance, underlining a critical need for integrated, locally informed approaches to pesticide management. This study also demonstrates the valuable role of extension officers in highlighting challenges related to pesticide use in farming communities and advancing research that engages with them.

## 1. Introduction

Pesticides are critical to agricultural productivity and food security, but with significant drawbacks to their widespread proliferation and use. Many pesticides have broad-spectrum effects that can harm non-target organisms and physical properties that facilitate long-term persistence and toxic bioaccumulation in food chains [1]. These problems are commonly associated with synthetic pesticides rather than biopesticides [2,3], although pesticides of all classes can decrease growth and reproduction across a wide range of animals, plants, and microbes [4]. Nonetheless, most research focuses on health hazards of synthetic pesticides, particularly among farmers, due to their dominant use in agriculture around the world [5]. Farmers’ exposure to these pesticides has been linked to many adverse effects including certain cancers, DNA damage, oxidative stress, neurological disorders, respiratory impairment, and thyroid dysfunction [6].

Although global pesticide use in agriculture has doubled since 1990, the regional trends are highly variable, with a 5% decline in Europe and a 185% increase in Africa [7]. Even with this increase, Africa accounted for only 5% of the global total and had the lowest levels of use reported by the Food and Agriculture Organization of the United Nations (FAO) [7]. However, lower pesticide does not necessarily mean lower health risks [8]. Studies in many African countries have reported limited pesticide-related knowledge and unsafe practices, in some cases with interventions to raise awareness about health risks and improve use of personal protective equipment (PPE) [9,10,11,12,13,14,15]. Notably, a survey of nearly 400 articles in 37 countries in sub-Saharan Africa identified several geographical hotspots for this research, along with some gaps, revealing an incomplete picture of pesticide risks for many ecosystems and populations in the region [16].

In this study, we held discussions with agricultural and animal resources officers about concerns and challenges related to pesticide use by Rwandan farmers, including effects on animals and ecosystems as well as human health. Our goal was to better understand their perceptions of pesticide-related health risks and knowledge among farmers in their sectors and potential differences between men and women. Research on this topic has been relatively limited in Rwanda in several respects, which we aimed to address with wide-ranging conversations and a unique range of perspectives. Most previous studies have focused on certain crops, pests, and/or regions [12,17,18,19,20,21,22], and with one exception [23], have lacked a One Health framework that emphasizes the shared threats of pesticide misuse to human, animal, and environmental health (for more information about One Health in general and in relation to pesticides specifically, see [24,25]). Women also have been underrepresented in samples and analyses in earlier studies [12,17,20]. Furthermore, an administrative perspective on pesticide use has been largely absent from scholarly publications (excluding “gray literature” such as reports produced by government agencies), despite the essential role played by local agronomists and veterinarians in supporting farmers and agricultural priorities in their districts. From a series of focus group discussions with sector officers in Rwanda’s Eastern Province, we conducted a thematic analysis and a pesticide assessment that highlighted potential drivers and dangers of pesticide use, exposure risks for people, livestock, and wildlife, and opportunities to facilitate safer pesticide practices.

## 2. Materials and Methods

### 2.1. Sample Background

The Rwandan government is organized into national and local spheres that regulate and promote agriculture and animal husbandry nationwide. The national government includes the capital city of Kigali and four provinces (Northern, Eastern, Southern, Western), led by governors who coordinate between the national and local governments. The Ministry of Agriculture and Animal Resources (MINAGRI) operates in the national sphere to establish policies and programs for agriculture and livestock, which are implemented by the Rwanda Agriculture and Animal Resources Development Board (RAB) [26]. Another one of MINAGRI’s development boards, the National Agricultural Export Board (NAEB), is responsible for exports of agricultural and livestock products [27].

Across Rwanda’s four provinces, the Ministry of Local Government (MINALOC) oversees local authorities across four top-down tiers of 30 districts, 416 sectors, 2148 cells, and 14,837 villages [28]. Each district and sector has an agronomist who is responsible for local implementation of agricultural activities, known as District Agricultural Officers (DAOs) and Sector Agricultural Officers (SAOs) respectively. Likewise, each district and sector has a veterinarian who is responsible for local management and development of animal resources, known as District Animal Resources Officers (DAROs) and Sector Animal Resources Officers (SAROs) respectively. These agronomists and veterinarians are extension officers who work in coordination with RAB and NAEB to support local farmers by providing extension services (e.g., advice, training, and distribution of resources such as seeds, fertilizers, and tools) and helping to achieve agricultural and livestock goals set by local government officials each year. Their efforts are reinforced by Socio-Economic Development Officers (SEDOs), who work at the cell level (a grouping of several villages) to coordinate farming activities and farmer promoters. Farmer promoters are volunteer community leaders trained by RAB and MINALOC to serve as farmer-to-farmer extension agents in their own villages. There is one farmer promoter in every village in Rwanda and thus more than 14,000 in total.

### 2.2. Sample Selection and Recruitment

This study was the first phase in a multi-year project to investigate use and potential health effects of agricultural pesticides in Rwanda. The formal research was preceded by an initial scoping conversation with five SAOs in Kayonza district of Eastern Province on 11 May 2024. This conversation was organized through mutual colleagues between one of the authors (Rwibutso) and the SAOs, who offered some informal insights into the use of pesticides by farmers in their sectors. These SAOs encouraged research on agricultural practices and health effects related to pesticide exposure, and they shared observations and anecdotes about topics of concern.

Based on this conversation, we designed a sampling scheme for a series of focus group discussions (FGDs) with SAOs and SAROs in Eastern Province. Our goal was to identify common practices and perceived challenges involving pesticides across the entire region, based on representative views from a range of districts and sectors. We also intended for these conversations to inform our direct engagement with farmers in the two remaining phases of our project, which are focused on data collection via questionnaires (Phase 2) and intervention via workshops (Phase 3). We thus chose to focus on SAOs and SAROs for recruitment, given their broad administrative perspectives on these topics and first-hand knowledge about pesticide use among farmers, as demonstrated in our scoping conversation. Their direct and daily interactions with farmers in support of agricultural and animal productivity, through hands-on training and one-the-ground guidance, differ from the activities of other technical officers in local government, such as Sector Environmental Officers (SEOs) (who focus on environmental protection) and DAOs and DAROs (who focus on regional policy implementation and coordination). Nonetheless, by focusing solely on SAOs and SAROs, we accepted a tradeoff of potential biases among the participants, which are discussed in Section Limitations about study limitations.

**Figure 1:**
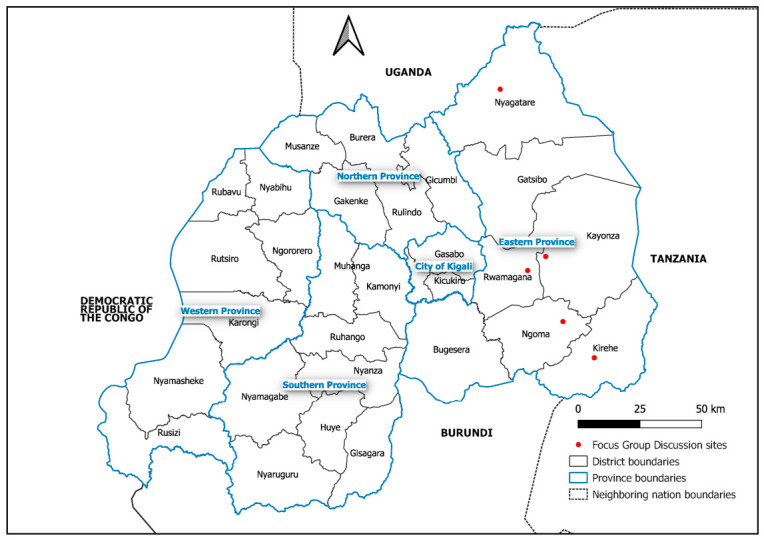
Map of Rwanda showing the locations of focus group discussions in the five selected districts. Printed from under a CC BY 4.0 license, with permission from Marie Aurore Ugirabe, original copyright 2025.

For participant recruitment, we employed the following approach during the period of 17–26 September 2024:

1. All seven districts of Eastern Province have similar diversity and intercropping patterns in four key crops (maize, beans, cassava, banana), with the exception of the recently populated Nyagatare district and a large part of Kayonza district containing Akagera National Park [29]. In determining the districts for recruitment, we prioritized thos with the highest levels of crop production and pesticide use [30]: Kayonza, Kirehe, Ngoma, Nyagatare, and Rwamagana (Figure 1).
2. Each of the prioritized districts is divided into 12 or 14 sectors, which we grouped by their location within five areas of the district: north, east, south, west, and center. We designed these groupings to capture variation in dominant crops and most commonly used pesticides between different areas of each district; for instance, given that four of the prioritized districts share borders with Uganda, Tanzania, or Burundi, farmers along these borders may be more likely to encounter pesticides that are less common in interior parts of Rwanda.
3. For focus group research, four groups are typically adequate to achieve code saturation (the point at which no additional themes are identified in data), whereas more are usually needed for meaning saturation (the point at which the identified issues are fully understood) [31]. Considering that our participant pool was demographically and occupationally homogenous, we anticipated that four or more FGDs would enable data and thematic saturation. In order to capture the full geography of the region, we opted to include an additional focus group, for a total of five, which is consistent with methodological guidelines [32,33]. In determining group size, we followed recommendations that around six participants would allow for a manageable, in-depth discussion in which each participant would have sufficient speaking time to address our questions and express their views [32,34,35]. We therefore recruited one SAO from one sector in each of the five areas within each prioritized district, as well as one SARO from one of the selected sectors to provide an animal health perspective on pesticide issues. The selected sectors are not listed here to protect the identities of the participants, as there is only one SAO and one SARO per sector.
4. We employed snowball sampling as a recruitment strategy for selecting specific participants. This network-based form of convenience sampling relies upon existing social connections to find and recruit participants, who are among the acquaintances of a small number of initial contacts [36]. For this study, the initial contacts were five SAOs from our scoping conversation, whose willingness to participate in a formal study was confirmed by Rwibutso. She delivered these invitations verbally via phone call, guided by a script that summarized the study and its objectives and provided details about the benefits, risks, and privacy protections associated with participation. Rwibutso also encouraged these initial contacts to suggest other potential participants who met the selection criteria of being a SAO or SARO from one of the prioritized districts and whose sector area was not yet represented in our sample. The individuals identified through this process were then invited to participate in the study by Rwibutso or another one of the authors (Nyirahabimana). Recruitment ended when at least six participants (five SAOs and one SARO) were confirmed for an FGD in each of the five selected districts.

### 2.3. Data Collection

After the Consent Forms were read and explained by the moderator of the FGDs (Rwibutso), they were signed by the participants and collected by our team. To protect their identities, participants then drew random numbers to be used in place of their names. They practiced responding to their number designation with the moderator before we activated an Aiworth 136 GB digital voice recorder to document the discussion for subsequent transcription, translation, and analysis. The voice recorder was positioned in full view of the participants, who (along with the Rwibutso and note takers Irimaso and Nyirahabimana) sat in chairs facing each other in a square or circular configuration. All of the discussions were conducted in Kinyarwanda and lasted for approximately two hours, following a guide of pre-written questions on six main topics: (1) general use of pesticides in agriculture, (2) specific uses of pesticides by farm workers, (3) different uses of pesticides by men and women, (4) animal exposure to pesticides, (5) potential health effects of pesticide use or exposure, and (6) priority concerns involving pesticides in agriculture. Specific questions, for example, included: “How does pesticide use today differ from the past?”, “Do you think there are any differences in how men and women agricultural workers perceive pesticide risks and safety behaviors?”, and “Are animals ever exposed directly to pesticides by farm workers, either intentionally or not?” The moderator concluded each FGD with an invitation for advice on engaging with farmers in future research and for comments on any topics not covered (for procedural details and the script outline, see Protocol S1 in the Supporting Information).

Researcher positionality and reflexivity are important considerations in qualitative research, particularly when discussions involve potentially sensitive topics such as public health [37]. Rwibutso’s professional background in veterinary medicine and basic knowledge about livestock pesticides may have eased technical conversations about pesticide use and animal health. Additionally, her familiarity with some of the participants or their colleagues may have facilitated rapport with them. Nonetheless, as a younger researcher outside of the participants’ administrative system, Rwibutso also prepared for reluctance by some participants to openly discuss pesticide misuse among farmers, perhaps due to concerns about professional accountability. To reduce these effects, she emphasized anonymity and confidentiality before the FGDs began and encouraged participants to share a range of perspectives and experiences openly.

### 2.4. Data Analysis

We transcribed the notes and voice recordings from the FGDs and translated these documents from Kinyarwanda into English. Each translator independently prepared an initial English translation of an assigned document (Rwibutso and Nyirahabimana for transcripts and Irimaso for notes) and then shared it with the others for review. The translators compared word choices, clarified ambiguous expressions, and addressed culturally specific terms or phrases that did not have direct English equivalents. Any challenging or uncertain elements were discussed until reaching consensus on the most appropriate English translation, and the final version of every document was proofread by Mukamana. This collaborative process facilitated consistency and accuracy across the documents, while also helping to preserve the intended meaning and nuances of participants’ responses for subsequent thematic analysis.

We conducted a deductive (or theoretical) thematic analysis of the English transcripts using Dedoose version 10.0.25 software. We used this type of qualitative analysis as a method for identifying patterns or themes within qualitative data that are relevant to a particular research focus or question, in contrast to an inductive approach with predefined categories or topics of discussion [38,39]. Following Braun and Clarke [38], we read the transcripts several times independently and discussed our initial ideas about potential themes and points of interest. We then generated initial codes for selected excerpts of text across the five transcripts, using Dedoose’s tools for real-time coding that enable multiple users to work on the same project simultaneously. These initial codes were descriptive labels for words or phrases of interest, and through a recursive process of refinement, many of them were grouped under broader codes that facilitated the identification of overarching themes. For example, in this hierarchical organization of data, child codes such as “climate change”, “new or more pests”, and “pest resistance” were grouped under the parent code of “increased pesticide use”, because they were all mentioned as factors or drivers of increased pesticide use. Participants’ comments about differences between men and women were analyzed as socially constructed characteristics of gender, with child codes such as “male daring” and “female caution” grouped under the parent code of “gender differences.”

All of the codes created for the code tree in Dedoose included short descriptions to facilitate their appropriate and consistent use throughout the transcripts. These descriptions were critical, as Dedoose is only an organizational tool whereas the analysis was performed by our team. Two coders (Mitchell and Sholts) generated and applied codes independently, and any disagreements upon comparison were resolved by discussing and clarifying code definitions or by deleting or merging codes that captured an unnecessary level of detail. These discussions were important for code reflexivity, as the coders had differing backgrounds and disciplinary expertise that influenced their focus on certain details, such as animal or disease descriptors. We determined thematic saturation at the point where all themes appeared consistently across all five focus groups and no new codes were added upon final review of the transcripts.

In addition to thematic analysis, we compiled a list of pesticides used by farmers in the five selected districts in order to evaluate their active ingredients. The list consisted of pesticides that were (1) named by participants when asked by the moderator about “types of pesticides most commonly used by farmers these days” and written on a flipchart by one of the note takers, (2) mentioned by participants during the FGD and extracted from the transcripts, or (3) observed and recorded during our visits to Agro&Vet shops that sell pesticides in each district. For observations at Agro&Vet shops, we visited at least one shop in each district with a team member fluent in Kinyarwanda, and we asked the shop workers to show us the pesticides sold there. We photographed each formulation we observed and noted the active ingredient(s) in each product.

### 2.5. Ethics Statement

This study received a determination of exempted research by the Smithsonian Institution Human Subjects Institutional Review Board (Protocol HS24043), and it was approved by the Smithsonian Privacy Office (Privacy Assessment 633376), the Office of the Director of Research and Innovation, College of Agriculture, Animal Sciences & Veterinary Medicine, University of Rwanda (ref 06/2024 DRI), and the mayors of the five districts where data collection took place.

## 3. Results

A total of 31 individuals participated in five FGDs in Kayonza, Kirehe, Ngoma, Nyagatare, and Rwamagana. Four of the FGDs consisted of six participants (five SAOS and one SARO), and one FGD had seven participants (six SAOs and one SARO). Every participant was over 18 years old, and most of them were men, due to male overrepresentation in these positions; at present, between zero and five (that is, 0% and 36%) of the SAOs in each of the five districts are women (personal communication from SAOs to Rwibutso). However, all but one of the FGDs had at least one female participant.

Four main themes were identified through our thematic analysis: (1) interconnected challenges, (2) shared exposure and health risks, (3) gender differences in risk behavior, and (4) transmission of knowledge. Below we summarize the most salient information for each theme, followed by the findings of the pesticide assessment. Each theme is supported by figures (and in one case a table) that report code frequencies, which are the number of times that different codes were applied within a single FGD or across all five of them. We use these code frequencies descriptively, in order to illustrate the relative prevalence and emphasis of certain topics across participants and discussions. However, they should be interpreted with caution, as the number of times a code appears does not necessarily reflect its significance or importance to participants.

### 3.1. Theme 1: Interconnected Challenges

Participants in all the FGDs agreed that pesticide use has recently increased in Rwandan agriculture, responding affirmatively to our question of whether pesticide use has changed over the past 10 years. One participant characterized the increase as “remarkable” and many of them explained various ways that pesticide use has grown over the last decade, from a time when farmers used few pesticides or only used natural insecticides such as ashes. For instance, we heard that “increase in population not only in Rwanda but also globally led to increase in demand for food, which has led to more people doing agriculture and greater pesticide use” and therefore “the number of people who use pesticides also increased.” Participants also told us that more people are growing plants that require pesticides, which are being applied in more ways (such as treating crops with pesticides prior to storage) at higher doses and frequencies, and with wider availability through local Agro&Vet supply stores. Additionally, numerous participants noted that the types of produce requiring pesticides have increased:

“*Chemicals are being widely used. In the past we knew that they were used in vegetables, fruits, and coffee…Now they are also used in maize and banana. It is difficult to find plants that are not sprayed with chemicals*.”

“*There has been change in pesticide use over the years, because there is increased pests and nearly every crop has pests, and it needs pesticide application*.”

The most commonly grown crops in each district were listed by participants, divided into staple crops (also known as subsistence crops, which are grown for household consumption and provide food security for Rwandan families) and cash crops (which are primarily grown for commercial purposes and contribute significantly to the economy through exports) (Table 1). Participants told us that some plants serve dual purposes as both staple and cash crops, such as banana in Kirehe and maize in Kayonza and Nyagatare.

**Figure 2:**
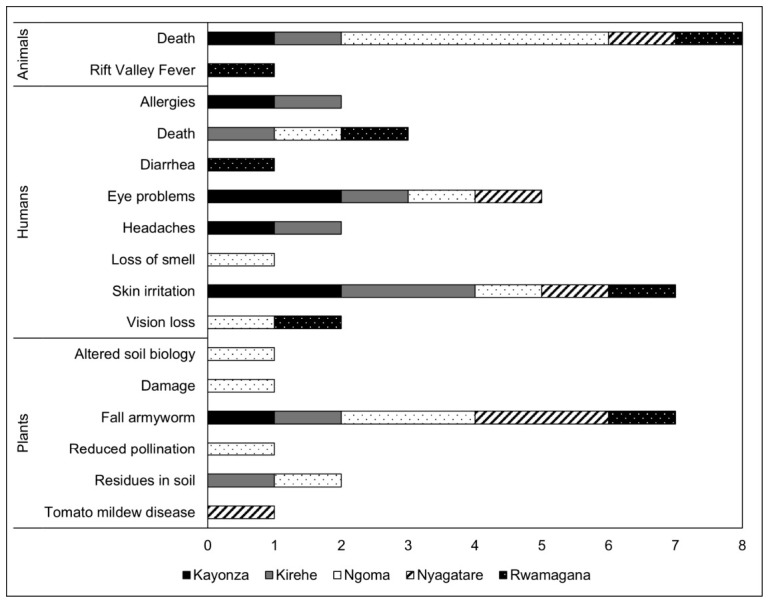
Code frequencies across all focus group discussions for human, animal, or plant health impacts of pesticides exposure. The number of participant responses corresponding to each code is shown by group and in total.

Maize was one of the plants named by participants in all of the FGDs as a primary staple crop in their districts. Maize is also the primary host of fall armyworm (*Spodoptera frugiperda*), a crop-damaging caterpillar species that was mentioned at least once in every group as both a cause and consequence of increased pesticide use (Figure 2). In several of these statements, participants made connections between climate change, pest resistance, and newly invasive insects:

“*In my observation, I think pesticide use has increased over the years mainly because of climate change, which has led to new pests’ emergence—for example, this one that heavily affects maize called fall armyworm*.”

“*Pesticides have recently come in maize due to armyworms and climate change. During the summer, there are many insects, but pesticides are being misused like mixing several chemicals at once in one container*.”

“*Some pests have developed resistance against some pesticides, and they will require another special treatment, for example fall armyworm*.”

**Figure 3:**
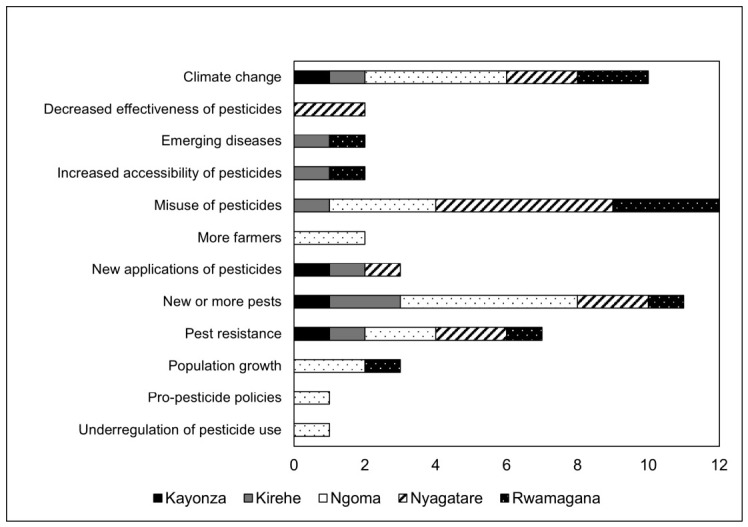
Code frequencies for causes of increased pesticide use across all focus group discussions. The number of participant responses corresponding to each code is shown by group and in total.

These problems were discussed in all the FGDs as reasons for increased pesticide use (Figure 3). Notably, we did not prompt these topics; they were raised by participants in response to an open-ended question about why pesticide use in Rwanda has increased in recent years. Fall armyworm was the only insect specified by name, just one example of the “many new pests” that participants believed that farmers have faced as a result of climate-driven invasion and field-evolved resistance. Recognizing the scale of these problems and the self-reinforcing process in which they are intertwined, participants spoke about how farmers have tried to mitigate their effects by using pesticides more intensively. As one participant described the situation:

“*It is clear that as time goes by, compared to the past, there are now pests that can cause farmers to lose everything they have grown, which is why they are resorting to using pesticides more often and even using higher doses so that resistant pests also die. There are also new pests that we have not encountered*.”

Additionally, the enormity and complexity of these problems were underscored by a sense of urgency among the participants, one of whom commented:

“*It is like we are sitting on a bomb. We in agriculture see it, but also people who do not do agriculture directly are also affected because they consume agricultural products*.”

### 3.2. Theme 2: Shared Exposure and Health Risks

A second theme of interconnectedness across the FGDs was shared pesticide exposure and health risks between people, animals, and plants. Although the term “One Health” was not said by any of the participants, their observations and anecdotes conveyed their understanding that human, animal, and ecosystem health are inextricably linked, which is not surprising given their educational backgrounds and professional expertise in these topics.

**Figure 4:**
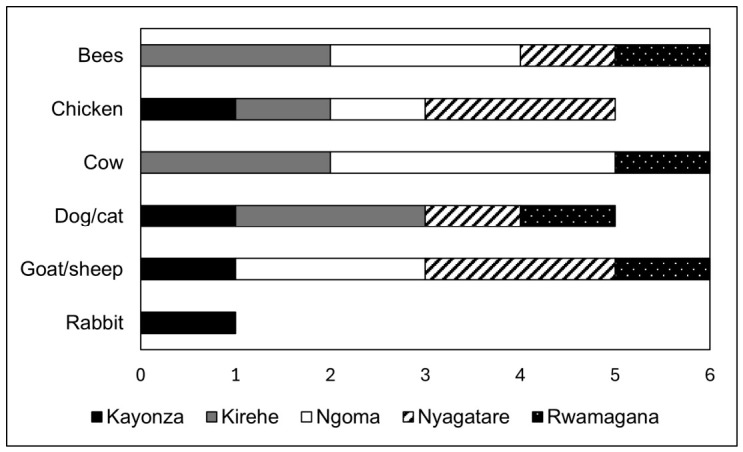
Code frequencies for animals exposed to pesticides across all focus group discussions. The number of participant responses corresponding to each code is shown by group and in total.

Animal deaths were mentioned in every FGD, both in reference to livestock such as cows, goats, and chickens as well as wildlife such as bees and other beneficial insects (Figure 2 and Figure 4). Participants in all groups spoke about the common occurrence of unintentional animal exposure to pesticides through consumption of pesticide-treated plants, particularly in the fields where animals were able to graze or roam and in the fodder (e.g., cut grass, maize, and other leftover crops) fed to them. One participant knew of cows dying after grazing in sprayed fields, and other participants brought up the health risks of contaminated cow’s milk due to pesticide residues in feed:

“*In Musanze, after Irish potatoes are sprayed with pesticides, the leftover plant material is usually fed to cows, resulting in milk with a pesticide-like smell. This shows how the pesticides residues may be transmitted to humans*.”

“*Some animals may succumb to pesticide exposure. Additionally, dairy farmers may face economic losses at the Milk Collection Center if their milk tests positive for residues and is subsequently rejected. Unfortunately, this rejected milk is often consumed by family members, including women and children, potentially leading to serious health risks*.”

Another comment about a water source contaminated with runoff from sprayed fields also illustrated the shared exposure between cows and people:

“*Sometimes animals like cows for example are exposed when they go to drink water, and that water gets mixed with water which came from fields where pesticides were applied. Sometimes even shepherds take a bath in that same water*.”

Furthermore, some statements emphasized the health connections between animals and plants, such as one answer to our question about potential human health risks from pesticide use in agriculture:

“*Other consequences include the loss of bees, which is associated with reduced pollination. Additionally, weed killers also eliminate other beneficial insects in the soil that are essential for crop health*.”

However, there was not a clear consensus among the participants on whether and how farmers perceived pesticides as a health threat to animals. In response to our question about farmers storing pesticide containers and application equipment in places where animals sleep, one participant in Rwamagana stated that “farmers know that pesticides are bad for animals, so they keep them away” whereas participants in Kayonza, Ngoma, and Nyagatare said that pesticides are commonly stored near animals in small and poor households in rural areas. Similarly, when asked about farmers intentionally exposing animals to pesticides, one participant in Rwamagana claimed that “no one can do it—if it happens, it might be an accident” and a participant in Kirehe opined that “I think farmers expose their animals unintentionally usually due to ignorance, poor living conditions, and sometimes negligence.” However, participants in Ngoma talked about farmers spraying plants with the intention of killing crop-raiding chickens and goats and fighting back against the robbery of grasses. Furthermore, in Nyagatare we were told that “No consideration is given to animals when spraying pesticides.” In all of these districts, participants spoke of livestock being treated frequently with pesticides to reduce ticks and during an epidemic of Rift Valley Fever to repel mosquitos.

Participants consistently expressed concerns about the health risks of pesticide exposure for farmers, based on conditions reported to officers or witnessed by them. As one participant expressed, “Anxiety is there because people are not safe.” Many of their concerns were related to misuses of pesticides by farmers, including improper handling, storage, and applications of pesticides and their equipment and containers. Participants also gave examples of potential human exposure from pesticide-treated produce, such as tomatoes and watermelons, and one participant recounted an incident of pesticide-treated sorghum having been used to make beer that caused illness and possibly death among the people who drank it. Other mentions of human deaths were related to the use of pesticides for suicide. Across all of the FGDs, participants recounted observing or being told by farmers about many incidents of sublethal effects of pesticide exposure, including skin irritation, loss of smell or vision, eye problems, headaches, diarrhea, and allergies (Figure 2), with comments such as:

“*We have received numerous reports of vision impairment, rashes, and allergies following the application of pesticides*.”

“*What I have come to notice is that usually reactions to pesticides appear a bit later after its application, sometimes the farmer doesn’t even connect the reaction to the pesticide application. Some reactions include skin itching, eye allergy, loss of sense of smell*.”

### 3.3. Theme 3: Gender Differences in Risk Behavior

We identified a third theme of variable risk behavior across the FGDs, based on many insights from participants about factors and motivations that influence pesticide exposure among farmers. In particular, they described some broad distinctions between men and women in actions and activities related to pesticides, and they expressed diverse opinions about how men and women differently perceive and manage their exposure.

There was a shared perception across the FGDs that nearly all the pesticide spraying of field crops—98–99% by some estimations—is done by men. “Sometimes a woman may fetch water and mix chemicals for him,” one participant explained, “but carrying the knapsack sprayer is for a man.” According to some participants, the small proportion of women who spray pesticides themselves are likely to be unmarried (e.g., young women, single mothers, and widows), although even women without husbands may turn to men for help. In contrast, participants stated that the main tasks performed by women are planting, weeding, and harvesting crops in the fields, followed by processing, sorting, and applying pesticides to the harvested crops for storage. However, it was noted that sometimes men and women may do some of this work together.

Despite the uniform descriptions of male-female divisions of labor, especially involving pesticide applications, there was considerable variability in how participants characterized protective measures taken by men compared to women. In general, they all observed a widespread lack of PPE use in farming in their districts, describing it as “low,” “uncommon,” and “almost non-existent” and with one assertion that “I have never seen even one farmer with full PPE.” Knowing that PPE is an important means of reducing pesticide exposure and health risks, the participants expressed concerns such as “We are worried because farmers do not protect themselves.” They told us that boots are the most frequently worn item of PPE, and often the only one, although masks are also sometimes worn. However, when we asked if men or women are more likely to wear PPE, their responses diverged. As shown in Table 2, participants in three of the FGDs responded that women wear PPE more than men, whereas participants in the other two FGDs said the opposite.

Participants attributed the underuse of PPE to a number of obstacles, such as PPE being too expensive or not sufficiently available for most people. Other speculations were that farmers did not have enough information or understanding about the importance of PEE for reducing pesticide exposure. Nonetheless, these obstacles do not fully explain the sex-based differences observed by the participants. Men were more often seen as having greater access to and more interest in information about pesticides, but women were more often seen as being especially sensitive to the potential harms of pesticide exposure. Additionally, participants were evenly split between perceiving men as having greater, lesser, or equal understanding of pesticide risks compared to women.

**Figure 5:**
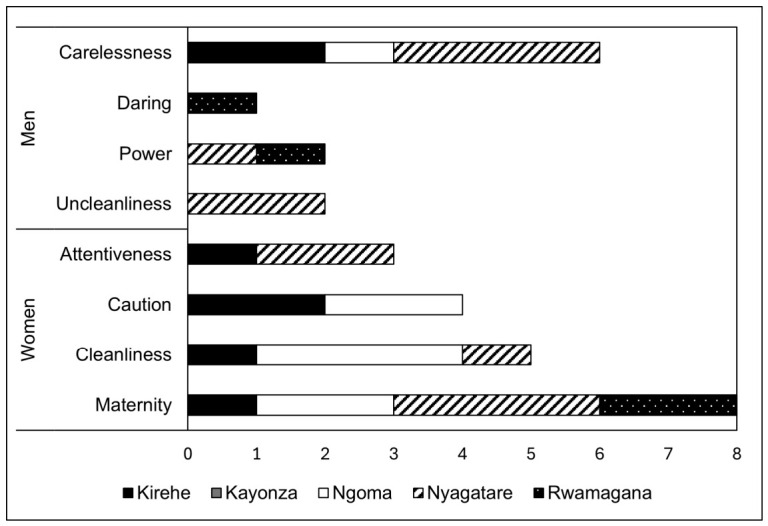
Code frequencies for characteristics attributed to men and women in all focus group discussions. The number of participant responses corresponding to each code is shown by group and in total.

The most consistent explanations of these differences were related to gendered characteristics that the participants ascribed to men and women. In Figure 5, these characteristics are listed by their codes and frequencies among the FGDs. In order of increasing frequency, female behaviors were associated with attentiveness, caution, cleanliness, and maternity, whereas male behaviors were associated with carelessness, daring, power, and uncleanliness. Many participants mentioned these attributes, and especially “maternity” with emphasis on pregnancy and motherhood in women, when discussing different actions and attitudes concerning pesticide risks:

“*Women often prioritize cleanliness and appearance, so even when involved in pesticide mixing or spraying, they tend to clean themselves and change clothes*.”

“*There are women who even carry knapsack sprayer. I advised her that if she continued working without PPE, she would never give birth again. She immediately protected herself. Men eat tomatoes with pesticides without even washing them. Men don’t care*.”

“*Women are more cautious than men usually due to their conditions like pregnancy, caring for young children. Some would not be physically able because they had some surgical operations done, and women care a lot about hygiene. Men have more information, but they don’t give much attention to the risks*.”

### 3.4. Theme 4: Transmission of Knowledge

The fourth theme that we identified in the FGDs is the importance of knowledge transmission in advancing safer and more effective pesticide use among farmers. Participants stressed that farmers are not the only ones who require more knowledge; people who advise farmers on pesticide use — including the extension officers themselves — also need to be better informed to help address pesticide-related challenges that are growing, multiplying, and evolving.

**Figure 6:**
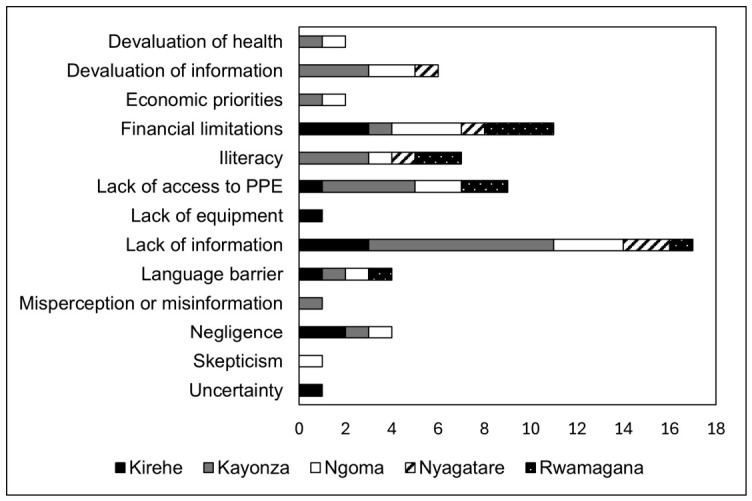
Code frequencies for obstacles to safer pesticide use in all focus group discussions. The number of participant responses corresponding to each code is shown by group and in total.

**Figure 7:**
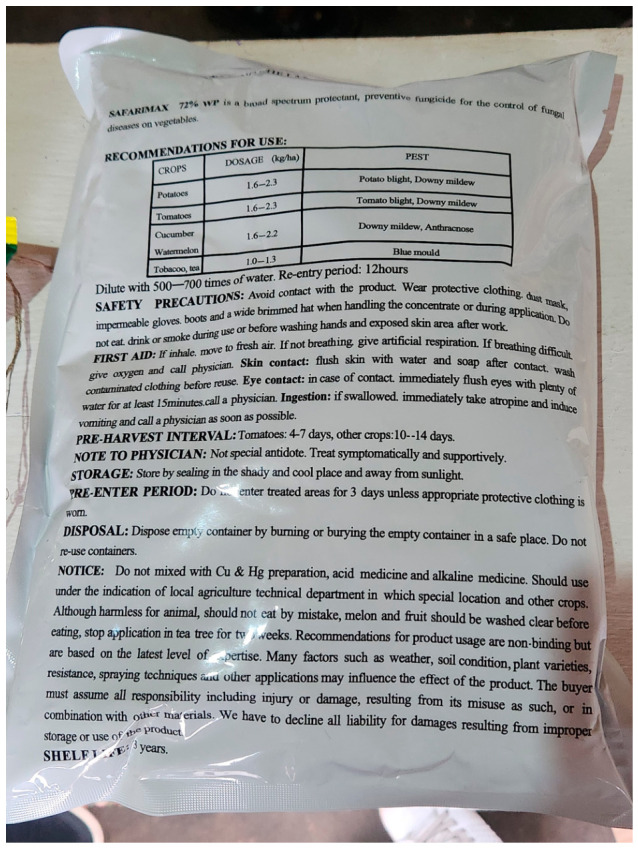
The label for a bag of mancozeb fungicide (SafariMax) that is commonly available in Agro&Vet stores in Rwanda. This wettable powder formulation requires dilution with water in specific quantities for applying to various crops with a sprayer. These recommendations, along with other instructions and health and safety precautions, are entirely in English. We frequently observed labels in numerous languages among the pesticides in these shops, as there is currently no legal requirement for labeling in Kinyarwanda [42]. Photo credit: Sabrina Sholts.

By far, the most frequently named obstacle to safer pesticide use by farmers was lack of information (Figure 6). For instance, participants pointed out that language barriers and illiteracy prevented farmers from being able to read the labels on many of the pesticides available to them, because the instructions and safety precautions are often in a language that most farmers do not know, such as English (Figure 7). There were numerous suggestions to improve labeling, such as “It would be better for the bottles to be labelled in Kinyarwanda and write on the bottle the consequences of the chemicals for people who do not comply with the instructions.” However, some participants also noted that many farmers do not have the education to understand these labels, even in a local language, and thus rely on the Agro&Vet dealer in the shop for guidance:

“*When pesticides are in bags or bottles, they usually have an etiquette which has instructions on how to use them. However, not everyone can read or understand these instructions, so they often ask the store for guidance on how to proceed*.”

Agro&Vet dealers and farmer promoters were most frequently identified as top sources of information about pesticides for farmers, second only to SAOs and SAROs. For this reason, the participants stressed their desire for more training for themselves, so that they can better serve farmers with up-to-date information about which pesticides to purchase and how to use them. As one participant explained:

“*There is a need for continuous training for people who work with farmers, such as agronomists and farmer promoters because pesticides are changing, even agrodealers do not have all the information they need. Additional information should be provided in addition to the information written on the bottle. The relevant agency should provide continuous training*.”

**Figure 8:**
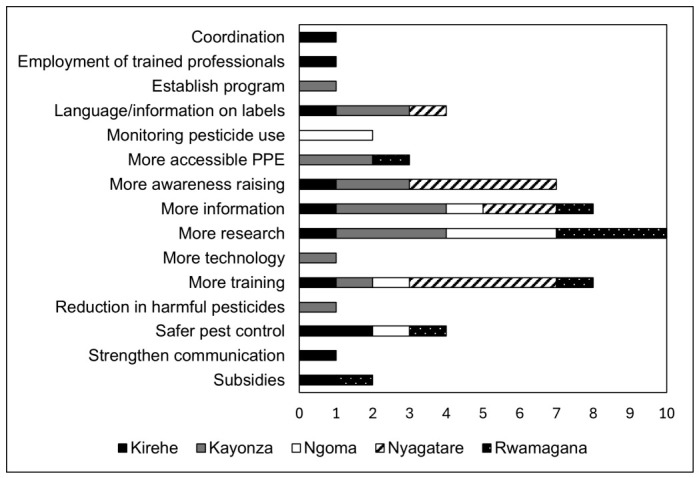
Code frequencies for changes needed for safer pesticide use in all focus group discussions. The number of participant responses corresponding to each code is shown by group and in total.

This recommendation was among many offered by participants when asked about what changes would be needed to improve pesticide use and safety in Rwandan agriculture. Across all focus groups, information and training were most frequently mentioned, followed by research (Figure 8). The participants saw many knowledge gaps where more research would be useful, covering a wide range of topics such as “pesticide resistance”, “the effectiveness of pesticides that no longer work in livestock farming or crop production”, “the evolution of pests due to climate change”, “effective biological control methods as alternatives to chemical pesticides”, “the immediate and long-term effects that can occur to someone who misuses pesticides”, and “farmers’ perceptions of pesticide application.”

Notwithstanding their emphasis on the importance of information for changes in pesticide use, some participants opined that farmers do not value and therefore disregard the information provided to them. This is why some of them recommended research about how farmers understand pesticides and their potential health effects. Also, some comments made clear that information alone could not solve problems in pesticide use, including:

“*Information is not enough; it requires skills, research. PPE is not close to the farmers. But does being close to the farmers mean that they buy it immediately? No. There is a need to improve information and impacts related to the use of chemicals. Stakeholders are needed to raise awareness*.”

### 3.5. Pesticide Assessment

We also aimed to identify the main pesticide active ingredients used in the Eastern Province, based on those listed and mentioned in FGDs and those observed in Agro&Vet shops. Our assessment produced a list of 56 compounds, seven of which were unknown names given by participants and could not be associated with any pesticide (“archimetrine”, “copi”, “tiamidor”, “decamix”, “parmapy plus”, “radomouri”, and “pesto-agro”). One compound that was mentioned was a chemical class that encompasses multiple active ingredients (“pyrethroids/pyrethrums”). Of the remaining 48 compounds, 15 were active ingredients and 33 were trade names. In Table S1, the trade names and active ingredients have been linked (see Supplemental Files). Some of the trade names that were provided did not associate with any of the named active ingredients; in these instances, we determined the appropriate active ingredient and included it in this table and this analysis. This resulted in identification of an additional 9 active ingredients, for a total of 24.

**Figure 9:**
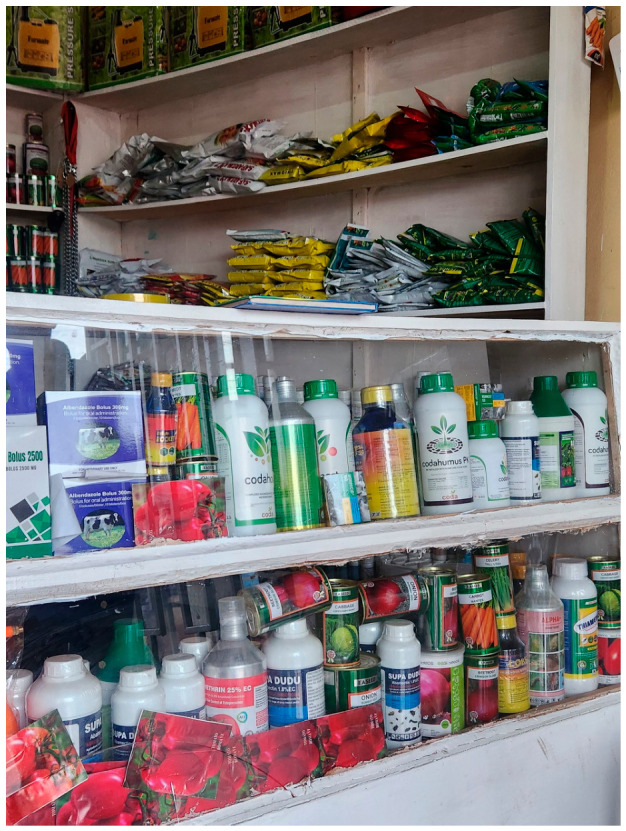
An Agro&Vet shop in Kirehe where farmers can buy pesticides for crops and livestock. Bottles of liquid formulations in the display case include abamectin (Supa Dudu, Dudu Aba+), cypermethrin (Ashimethrin 25% EC, Alpha+), profenofos-cypermethrin combinations (Rocket, Rocket 44EC), and thiamethoxam (Thiamedor). Bags of wettable powder formulations on the shelves include mancozeb-metalaxyl combinations (SafariMax) and thiamethoxam (Thiomax). Photo credit: Sabrina Sholts.

Of these 24 active ingredients, we note that two are no longer in common use and were discussed in other contexts. For example, Thiodan (active ingredient: endosulfan) was discussed as a pesticide that is now illegal and was only mentioned in the context of a suicide, and Vital (active ingredient: imidacloprid) was discussed in the context of a single acute poisoning event that led to blindness. Sixteen of the active ingredients (or formulations including those ingredients) were only mentioned in one or two districts and were not commonly observed in Agro&Vet shops. Six active ingredients (as well as multiple formulations including these ingredients) were reported in all five focus groups and observed in Agro&Vet shops in all five districts: abamectin, cypermethrin, lambda-cyhalothrin, profenofos, mancozeb, and metalaxyl (Figure 9, Table 3 and Table S1). These six active ingredients include both insecticides and fungicides and represent a very small fraction (less than 4%) of the more than 150 active ingredients registered for use as pesticides in Rwanda [40,41].

## 4. Discussion

The participants in the FGDs shared similar observations on a wide range of topics, which is notable considering the large number of farmers within their districts. Although Rwanda is the smallest East African country by population, it has the highest population density on the continent [43]. In the most heavily populated Eastern Province, there are more than 3.5 million residents, almost 70% of whom live in the five districts in this study [44]. The participants estimated that the proportion of farmers in these districts range from 65 to 80% in Kirehe to 99–100% in Ngoma, amounting to at least 2 million people under discussion (Table 1). However, it is critical to emphasize that the results of the FGDs reflect the participants’ perspectives alone and do not constitute direct evidence from the farmers themselves.

The increasing pesticide use described by participants is consistent with recent trends across Africa [7] as well as national agricultural statistics from Seasonal Agricultural Survey (SAS) reports. Conducted annually by the National Institute of Statistics in Rwanda (NISR) in collaboration with MINIAGRI, NAEB, and RAB and other organizations, the SAS differentiates farmers with more than 10 hectares of land (large-scale farmers, or LSFs) and those with less (small-scale farmers, or SSFs). Additionally, SAS data are collected separately for Rwanda’s three growing seasons: Season A (September to February) and Season B (March to June) are similar in their primary production of staple crops such as maize, whereas Season C (July to September) is used to supplement household diets with crops such as potatoes and vegetables [45]. Comparing the first SAS report to the most recent one, pesticide use by LSFs during Seasons A and B more than quadrupled from 17 to 20% in 2013 to 74–83% in 2024 [46,47]. The increase has been even more dramatic among SSFs, which dominate the agricultural sector, ballooning from 6 to 8% to 30–40%. Season C data are not consistently reported between SAS reports, and neither are data at sub-national levels, which make other changes less clear. However, a similar pattern can be inferred for Eastern Province, where only 7–12% of LSFs and 2–6% of SSFs used pesticides during Seasons A and B in 2013. In contrast, LSFs and SSFs in the five districts in this study had use rates of 60–100% and 12–37% respectively in 2024. These data support the “remarkable” increase observed by FGD participants in their districts and sectors over recent years. Interestingly, a study of farmers in Northern and Western Provinces published in 2019 found that none of them applied chemical pesticides to bananas [12], but our participants, asked 6–7 years later, stated that synthetic pesticides are now used on this crop.

Concerns about climate change pervaded the FGDs, likely related to the fact that Eastern Province is already the hottest region in Rwanda. Over the last several decades, Eastern Province has experienced accelerated warming throughout all growing seasons, especially in the northwestern and southeastern zones where the districts of the FGD participants are located [48]. Under future climate change, Eastern Province is estimated to suffer the most severe declines in potential crop yields of anywhere in Rwanda, especially in the vital staple crops of maize, bush bean, and Irish potato [45]. Apart from endangering crop productivity, climate change may also allow the invasive fall armyworm to become a permanent menace to farmers. Future climate projections suggest that global areas with climatic suitability for fall armyworm establishment will shrink over time, due mostly to heat and dry stress, yet countries with sub-tropical wet and dry climates—like Rwanda—will continue to offer optimal conditions for long-term persistence [49]. In such a scenario, Rwanda could serve as an overwintering area and source of seasonal invasions of fall armyworm to other parts of Africa for many years to come.

Pesticides are the main defense against fall armyworm for many farmers, which all FGD participants discussed with apprehension. First reported in West Africa in 2016, these highly polyphagous insects were identified in every district in Rwanda by 2017 [50,51]. As part of a study of SSF responses to fall armyworm in five African countries, Tambo et al. [20] surveyed 637 maize-producing households in Rwanda whose farms were attacked by fall armyworm in 2017–2018. While none of these households were in Eastern Province, the responses were consistent with our FGD results. Tambo and colleagues found that 87% of the Rwandan households used synthetic pesticides against fall armyworm, a much higher rate than farmers in other African nations in their study, possibly related to greater access to free or subsidized farm inputs. Indeed, in 2017 the RAB began to provide insecticides and equipment to Rwandan farmers with limited means for quick intervention against fall armyworm, and the Rwanda Defense Force delivered and replenished insecticides rapidly using helicopters [51]. They provided mainly formulations of profenofos and cypermethrin, which was used by 92% Rwandan farmers surveyed by Tambo et al. and shown to be most popular across East Africa for fall armyworm control. The pesticides used in the fall armyworm outbreak showed varying efficacy in killing the target insects, with most farmers in one study reporting a “good” status for their maize fields after pesticide applications in 2017 [52]. However, the effects of this spraying on nontarget organisms (such as pollinators and natural predators of crop pests) and the environment have not been documented [53].

Profenofos and cypermethrin were among the six active ingredients identified in all the FGDs as frequently used by farmers in Eastern Province. They are commonly used together in formulations sold under the trade names “Cypro 44EC”, “Profex”, “Profex Super”, “Rocket” and “Roket” (Table 3). While the labels and the adjuvants included in these formulations may differ, they all contain a mixture of 40% profenofos and 4% cypermethrin. As described under Theme 1, many of our study participants alleged that farmers often mix multiple pesticides together, with a goal of creating a more effective mixture. If common products like Cypro 44EC and Rocket are mixed together, this unfortunately does not result in a tool that targets multiple mechanisms of toxic action but simply increases the application of these two active ingredients above recommended levels.

Profenofos and cypermethrin were also among the many pesticides assessed by Jepson et al. [54] for risks to human, animal, and environmental health, PPE requirements, and efficacy against fall armyworm. They classified both of these pesticides as “high risk” and “requiring maximum PPE”, with efficacy against fall armyworm of “poor-to-fair” (<70–<80% control) for profenofos and “good-to-excellent” (80–100% control) for cypermethrin. When considered alongside participants’ claims of unsafe pesticide practices and limited PPE use among farmers, these classifications may suggest scenarios of likely overexposure, albeit without direct evidence within this study. In contrast, in the same efficacy range, there are at least 10 lower risk pesticides that require only single-layer PPE. Furthermore, these lower risk pesticides have diverse modes of action, and therefore rotating their use could limit selection pressure for insecticide resistance in fall armyworm, a point of anxiety for many FGD participants and a growing topic of research around the world [55,56,57,58].

Of the six commonly used active ingredients in Eastern Province, four (profenofos, cypermethrin, abamectin, and lambda-cyhalothrin) are neurotoxic insecticides. Cypermethrin and lambda-cyhalothrin are part of the pyrethroid class of chemicals, profenofos is an organophosphate insecticide, and abamectin is in the avermectin family. Exposure to these specific insecticides can lead to skin rash, eye irritation and vision problems, headaches, nausea and vomiting, respiratory issues and—at high exposures—tremors, seizures and even death [59,60,61,62]. Many of these symptoms were mentioned by FGD participants, as noted under Theme 2, as well as in previous research [12]. These individual active ingredients are sold in formations with a variety of labels and trade names (e.g., lambda-cyhalothrin is sold as Jackmax, Lambda, Ramda, and Lambda+; cypermethrin alone is sold as Alpha+, Simba+, and Ashimetrin, in addition to as a mixture with profenofos; abamectin is sold as Dudu, Dudu+, Supa Dudu, and Dudu Aba; see Table 2). Importantly, multiple names for a small number of active ingredients and formulations may increase resistance in target organisms, if farmers falsely assume they are using distinct pesticides. This may be especially true for fall armyworm, which has shown resistance to pyrethroids and organophosphates [58].

The other two commonly used active ingredients were the fungicides mancozeb and metalaxyl (also known as menfenoxam). Mancozeb is sold as a wettable powder as “mancozeb” but also under the trade names Mancobex, Dithane, Indothane, and Agrothane P+. Its fungicidal mechanism of action is not directly relevant to human health, and aside from skin irritation, acute human toxicity of mancozeb is low. However, numerous studies show mancozeb to be a reproductive hazard for humans, leading to decreased sperm count and motility, testicular damage, and male infertility [63,64]. Due to these concerns, mancozeb is not approved for use in the EU [65]. Metalaxyl is also a fungicide, primarily used to control mildew and late blight, and it is most commonly sold in combination with mancozeb, as the formulations Radomol and Ridomil. Metalaxyl is also thought to have low acute toxicity, though it is a known eye irritant.

According to REMA in their National Integrated Pest Management Framework for Rwanda, fungicide use comprises 75% of all pesticide use in Rwanda, insecticides comprise 23% and herbicides constitute the remaining 2% [66]. Our observations and findings were consistent with this report. While just two of the six most commonly used active ingredients were fungicides, these two compounds (mancozeb and metalaxyl) were mentioned multiple times in all focus groups and observed in numerous formulations and large quantities in every Agro&Vet shop we visited (Figure 9). Previous researchers have noted that farmers rely so heavily on mancozeb and metalaxyl that one in ten farmers use these fungicides in attempts to control the fall armyworm, even though the armyworm is not a fungus and cannot be impacted by these chemicals [21]. Consistent with the government report, herbicides were rarely reported or observed in this study, with hand-weeding by women being the most commonly reported method of weed management.

Above all else, the FGD participants emphasized the need for more information, research, and training to promote changes in pesticide use and risk behaviors among Rwandan farmers. For the SAOs and SAROs, this increased knowledge would help with guiding, assisting, and supporting farmers with pest control strategies in safer and more effective ways. For farmers, it could motivate safer practices, although financial limitations and accessibility are strong factors in pesticide selection and PPE use as well. For instance, in Eastern Province, Tambo et al. [22] have shown that information campaigns can be effective in enhancing farmers’ knowledge on how to identify and sustainably manage fall armyworm. In a campaign carried out in partnership with the RAB, they found that farming households exposed to mass-extension information channels (plant health rallies, radio drama, and text messages) were more likely to adopt integrative pest management (IPM) practices for fall armyworm, such as regular monitoring of maize fields and cultural, mechanical, and chemical control methods that reduce reliance on synthetic pesticides. Moreover, all the campaign channels were associated with significant increases in maize yields and incomes, suggesting IPM practices could have positive effects on maize productivity while reducing pesticide-related health risks. These findings highlight the potential benefits for SAOs, SAROs, and farmers in training focused on IPM, an ecosystem-based and data-driven approach to pest control and prevention, particularly given the importance of practical, locally relevant, and economically viable IPM methods for combating fall armyworm in sub-Saharan Africa [67].

Participants in this study expressed opinions about some gender differences in how farmers experience, manage, and understand pesticide-related health risks, consistent with many studies throughout sub-Saharan Africa [68]. Differences in pesticide exposure resulting from gendered divisions of labor, such as men being primarily responsible for pesticide application, have been reported in Ethiopia [69], Ghana [70], Kenya [71], Mali [72], Sierra Leone, [73], Tanzania [74], and Uganda [75]. Some participants in our FGDs described similar divisions among farmers in their districts, i.e., that pesticide spraying was done mostly by men and other tasks like weeding by women. In other African countries, researchers have also found that women typically have less knowledge of the harmful effects of pesticides and less access to IPM trainings [72,75], although as noted by Christie et al. [72], country-specific gender roles and power dynamics influence these differences. In Mali, for instance, they have suggested that female farmers may have fewer benefits or less interest in IPM programs compared to their male counterparts, because women are significantly less likely than men to be literate and to control the income earned from selling products that they farmed [72]. In our study, the FGDs did not convey clear gender disparity in decision-making power, education, and access to resources among Rwandan farmers, perhaps because Rwanda by many measures is one of the most gender-equal societies in Africa and around the world [76]. Nonetheless, in most of the FGDs, the participants described gendered characteristics that may affect how Rwandan farmers interact with pesticides. Women were observed as caring most about the potential health effects of pesticides on children and pregnancy, such as by limiting their exposure to reduce risks of infertility, whereas men were described as less concerned about pesticide-related health risks in general. By reporting these participant perceptions, we do not intend to reinforce or normalize gender stereotypes that possibly misrepresent the diverse experiences and behaviors of farmers. Rather, these findings highlight how socially constructed gender norms may shape perceptions and practices related to pesticide use in this context. This topic and others related to gender differences in pesticide-related attitudes and behaviors would benefit from further study, given that such information on Rwandan farmers is sparse and the findings could be helpful in developing and implementing effective interventions.

Although participants generally did not mention observations or knowledge about wildlife exposure to pesticides, bees were a notable exception. Expressed in almost every FGD, their concerns signify an ecological crisis in Rwandan agriculture, where massive honeybee collapse has been attributed to widely used pesticides such as Rocket [77]. This situation is consistent with the findings of our pesticide assessment that many products contain lambda-cyhalothrin and cypermethrin, which have been demonstrated as highly toxic to honeybees in pyrethroid mixtures [78,79]. Many FGD participants also spoke about pesticide exposure in cattle, goats, chickens, and dogs, mostly in reference to animals that died as a result of pesticide poisoning. Anecdotes about pesticide contamination in cow’s milk, such as people drinking milk that smells like pesticides, suggest health risks for these animals as well as the people who consume their products. Although unverified by direct evidence, these comments are noteworthy, because in many ecosystems, livestock and wildlife share access to grazing areas, water sources, and other ecological spaces [80]. Indeed, a recent study of rice farmers in Nyagatare district found that all of its participants had directly observed or had heard reports of cows, birds, or fish showing signs of pesticide intoxication (such as death, uncoordinated movement, and weakness) within 30 min to six hours after consumption of pesticides or presence in recently sprayed areas [17]. Almost half of those participants also reported seeing small fish die a few minutes after applying the pesticides, especially during plot preparations before planting rice, suggesting a link to pesticide-contaminated water.

Monitoring pesticide exposures in domestic animals may offer an early indication of threats to sympatric wildlife, especially in areas where direct wildlife monitoring is limited [81,82]. This may be particularly impactful in wildlife movement corridors, where frequent overlap between livestock and wildlife increases the potential for shared exposure to environmental hazards. Monitoring livestock exposure in these movement corridors could possibly help identify contamination hotspots and inform targeted mitigation strategies within a broader One Health approach [83,84,85]. However, the proposition that livestock exposure can be used as a proxy for wildlife exposure is largely speculative in this context. In general, data from domestic animals should be interpreted with caution, as their behaviors, diets, and potential for direct pesticide exposure may differ significantly from those of wildlife. While they offer important insights, domestic animals are best viewed as one component of a broader surveillance strategy that also includes direct wildlife monitoring and environmental sampling to fully assess pesticide risk.

### Limitations

There are several limitations to this study that should be considered when interpreting the findings. First, the FGDs included only SAOs and SAROs in Eastern Province. Although the participants represented a large proportion of the agricultural and veterinary extension officers within the five selected districts, their comments in the FGDs reflect their own perceptions, experiences, and professional interpretations rather than direct accounts from the farmers they serve. As local government officials who regularly interact with farming communities, the participants’ insights may have been shaped by their institutional responsibilities, training, and assumptions about farmers’ behaviors. Consequently, this study captures administrative and extension service perspectives on pesticide use and health risks, which may differ from farmers’ own understandings, motivations, and lived experiences. Our decision to focus exclusively on SAOs and SAROs also limited the diversity of administrative viewpoints represented in the discussions, as the findings may disproportionately reflect frontline extension priorities and observations.

Our sampling strategy combined convenience and snowball sampling, which may have introduced selection bias. Participants recommending colleagues within their own professional and institutional networks could have contributed to homogeneity of viewpoints, experiences, and professional norms. Officers who were more engaged, accessible, or interested in pesticide-related issues may also have been more likely to participate in the FGDs, potentially limiting the diversity of opinions captured. While this approach was practical for identifying willing participants within the selected districts and sectors, we acknowledge the possibility that the FGDs were skewed towards interconnected professional perspectives rather than the full range of extension officer experiences and knowledge about pesticide use among the farmers in their sectors. In particular, those SAOs who were willing to participate and who were well-connected within their professional networks might be those with greater awareness of, or concern about, pesticide exposures.

Given that the FGDs relied on participants’ recollections and interpretations, the findings may be influenced by their ability to recall observations. Participants may have unintentionally overstated or understated farmers’ pesticide practices, knowledge, or risk perceptions based on memorable events and personal experiences. Because discussions occurred in group settings, the FGD dynamics may also have introduced social desirability bias and thus influenced participation and responses. Some participants may have deferred to more outspoken or experienced colleagues, potentially limiting the expression of dissenting or minority perspectives.

Due to the majority of men among the participants, male perspectives were dominant in the FGDs, although this disparity was by design: gender balance in these discussions would not accurately represent the gender imbalance in these extension officer positions. Women’s perspectives may nevertheless have been underrepresented in the FGDs. In any case, regardless of the gender of the participants, their statements about gender differences among farmers, ranging from factors of pesticide use to exposure risks and safety behaviors, should be treated with caution unless validated with information provided by the farmers themselves.

The geographic scope of this study is another potentially limiting factor in this research. Rwanda is a small country where agricultural systems, pesticide use patterns, and environmental conditions do not vary widely between most regions. However, there are some significant differences in elevation, temperature, and vegetation between Eastern Province and the high-altitude volcanic mountains of the Northern Province, such that these findings may not be generalizable to all other regions of Rwanda.

It should be emphasized that the code frequencies reported in this study do not necessarily indicate the prevalence or importance of these issues across all farming communities or extension officers. These frequencies are useful for identifying recurring patterns and salient concerns, but they should not be interpreted as statistical measures of magnitude or representativeness.

Importantly, it was not the goal of this study to produce recommendations for policies or interventions based on the FGD results, as these kinds of determinations will be based on research in which farmers can describe their behaviors, perceptions, and concerns using their own words and voices. The results of this study therefore provide guidance for subsequent steps in the broader research project that directly engages with farmers and analyzes their views, along with considerations of quantitative research on animal and/or environmental health risks in these areas.

## 5. Conclusions

This study examines potential drivers, factors, and health risks of pesticide use in Rwandan agriculture through the wide-angle lens of sector-level officers in Eastern Province. Due to the professional positions of the study’s participants, the FGDs provided informed perspectives and broadly relevant information about a range of issues. We identified themes of perceived connections between increased pesticide use and other ecological challenges, observations of shared pesticide exposure between human, animals, and ecosystems, varying views on gender differences in risk behavior related to pesticide use, and the recognized importance of transmitting knowledge about pesticide-related threats as they emerge and evolve. We also determined that a large number of commonly used pesticides in Eastern Province contain only a small number of active ingredients, potentially contributing to a risk pathway for pest resistance. Notably, the FGDs emphasized a clear need and desire for more training on pesticide-related health risks and safety behaviors in Rwandan agriculture.

This study lays essential groundwork for research that engages directly with farmers and investigates potential sources of pesticide contamination and exposure in wildlife as well as livestock. Its findings may result in promising opportunities for public education and policy change to promote risk reduction involving pesticides, such as IPM training and pesticide safety workshops. Furthermore, the insights and information provided by the participants highlight how extension officers are a critical link between researchers and farmers in Rwanda, particularly on topics of pesticide use and health, and should be integrated more widely into research plans and activities.

## Figures and Tables

**Table 1:** Information provided by participants about the proportion of farmers in their district populations, the proportion of staple versus cash crops, and the main crops grown.

District	% Farmers	% Staple/Cash	Staple Crops	Cash Crops
Kayonza	85–99%	90 vs. 10%	maize, rice, cassava, beans, soybeans, sweet potato, peanut	coffee, macadamia, chili pepper, French beans, avocado, patchouli, flowers, maize
Kirehe	65–80%	More staple	maize, cassava, beans, sweet potato, banana	coffee, French beans, pineapple, avocado, banana
Ngoma	99–100%	80 vs. 20%	maize, beans, tomato, potato, banana, watermelon, pineapple	coffee, chili pepper, pineapple, avocado
Nyagatare	75–98%	90 vs. 10%	maize, rice, cassava, beans, soybeans, vegetables, potato	maize, coffee, pepper, French beans, mango, banana, avocado
Rwamagana	80–90%	Mostly staple	maize, rice, millet, yams, cassava, beans, soybeans, tomato, banana	coffee, macadamia, chili pepper, bell pepper, watermelon, pineapple, avocado

**Table 2:** Code frequencies for gender differences in pesticide knowledge, interest, safety behavior, understanding, and sensitivity in focus group discussions in Kayonza (KZ), Kirehe (KH), Ngoma (NM), Nyagatare (NT), and Rwamagana (RW). The number of participant responses corresponding to each code is shown by group and in total.

Codes	KZ	KH	NM	NT	RW	Total
**Access to pesticide information**						
Men have more access than women	1	2	1	0	0	4
Women have more access than men	0	0	0	0	0	0
No difference between men and women	0	0	0	0	0	0
**Interest in pesticide information**						
Men have more interest than women	2	1	0	0	1	4
Women have more interest than men	0	0	0	1	0	1
No difference between men and women	0	0	0	0	0	0
**Sensitivity to pesticides**						
Men are more sensitive than women	0	0	0	0	1	1
Women are more sensitive than men	0	0	2	4	1	7
No difference between men and women	0	0	0	0	0	0
**Understanding of pesticide risks**						
Men have more understanding than women	0	0	0	0	2	2
Women have more understanding than men	0	0	0	1	1	2
No difference between men and women	0	0	0	2	0	2
**Use of PPE**						
Men wear PPE more than women	1	0	0	0	1	2
Women wear PPE more than men	0	2	2	1	0	5
No difference between men and women	0	0	0	0	0	0

**Table 3:** Six active ingredients commonly used by farmers in Eastern Province. Listed by category, chemical class, and trade names, these pesticides were mentioned in all five focus group discussions and observed in Agro&Vet shops in all five districts.

Category	Chemical Class	Active Ingredient	Trade Name
Insecticides	Pyrethroids	Cypermethrin (including alpha- and beta-) ^a^	Alpha +
			Simba+
			Ashimetrin
		Lambda-cyhalothrin ^a^	Jackmax
			Lambdex
			Ramda
			Lambda +
	Avermectins	Abamectin ^a^	Dudu Aba+
			Supa Dudu
			Dudu
			Dudu +
	Organophosphate and Pyrethroid	Mix of Cypermethrin and Profenofos ^a^	Cypro 44EC
			Profex
			Profex Super
			Rocket
			Roket
Fungicides	Dithiocarbamate	Mancozeb ^b^	Mancobex
			Dithane
			Indothane
			Agrothane P+
	Dithiocarbamate and Phenylamine	Mix of Mancozeb and Metalaxyl ^a^	Radomol
			Ridomil

^a^ All four insecticidal active ingredients (cypermethrin, lambda-cyhalothrin, abamectin and profenofos) and metalaxyl are classified by the World Health Organization as Class II (Moderately Hazardous) pesticides [41]. ^b^ The fungicide mancozeb is classified by the World Health Organization as Hazard Class U (“unlikely to present acute hazard in normal use”) due to low acute oral and dermal toxicity.

## Data Availability

Raw data in the form of interview transcripts cannot be shared publicly to protect the privacy of the study participants. Data requests may be sent to the Smithsonian Institutional Human Subjects Institutional Review Board (researchcompliance@si.edu).

## Supplementary Materials

The following supporting information can be downloaded at: https://www.mdpi.com/article/10.3390/ijerph23060771/s1, Table S1: A complete list of active ingredients mentioned in focus groups and observed in Agro&Vet shops in all five districts.; Protocol S1: The protocol used for all five focus group discussions, including the introductory script and questions guideline.

## Abbreviations

The following abbreviations are used in this manuscript:

FAO	Food and Agriculture Organization of the United Nations
FGD	Focus group discussion
DAO	District Agricultural Officer
DARO	District Animal Resources Officer
IPM	Integrated pest management
LSF	Large-Scale Farmer
MINAGRI	Ministry of Agriculture and Animal Resources
MINALOC	Ministry of Local Government
NAEB	National Agricultural Export Development Board
PPE	Personal Protective Equipment
RAB	Rwanda Agriculture and Animal Resources Development Board
SAO	Sector Agricultural Officer
SARO	Sector Animal Resources Officer
SAS	Seasonal Agricultural Survey
SEDO	Socio-Economic Development Officer
SEO	Sector Environmental Officer
SSF	Small-Scale Farmer

## References

[1] Carvalho F.P. (2017). Pesticides, environment, and food safety. Food Energy Secur..

[2] Chandler D., Bailey A.S., Tatchell G.M., Davidson G., Greaves J., Grant W.P. (2011). The development, regulation and use of biopesticides for integrated pest management. Philos. Trans. R Soc. Lond. B Biol. Sci..

[3] Daraban G.M., Hlihor R.-M., Suteu D. (2023). Pesticides vs. biopesticides: From pest management to toxicity and impacts on the environment and human health. Toxics.

[4] Wan N.-F., Fu L., Dainese M., Kiær L.P., Hu Y.-Q., Xin F., Goulson D., Woodcock B.A., Vanbergen A.J., Spurgeon D.J. (2025). Pesticides have negative effects on non-target organisms. Nat. Comm..

[5] Damalas C.A., Koutroubas S.D. (2016). Farmers’ exposure to pesticides: Toxicity types and ways of prevention. Toxics.

[6] Curl C.L., Spivak M., Phinney R., Montrose L. (2020). Synthetic pesticides and health in vulnerable populations: Agricultural workers. Curr. Environ. Health Rep..

[7] FAO (2024). Pesticides Use and Trade—1990–2022.

[8] Williamson S., Ball A., Pretty J. (2008). Trends in pesticide use and drivers for safer pest management in four African countries. Crop Prot..

[9] Anaduaka E.G., Uchendu N.O., Asomadu R.O., Ezugwu A.L., Okeke E.S., Ezeorba T.P.C. (2023). Widespread use of toxic agrochemicals and pesticides for agricultural products storage in Africa and developing countries: Possible panacea for ecotoxicology and health implications. Heliyon.

[10] Mengistu D.A., Geremew A., Tessema R.A. (2024). Pesticide safety practice and its public health risk in African regions: Systematic review and meta-analysis. BMC Public Health.

[11] Ndayisaba F., Guo H., Bao A., Guo H., Karamage F., Kayiranga A. (2016). Understanding the spatial temporal vegetation dynamics in Rwanda. Remote Sens..

[12] Okonya J.S., Petsakos A., Suarez V., Nduwayezu A., Kantungeko D., Blomme G., Legg J.P., Kroschel J. (2019). Pesticide use practices in root, tuber, and banana crops by smallholder farmers in Rwanda and Burundi. Int. J. Environ. Res. Public Health.

[13] Rodenburg J., Johnson J.-M., Dieng I., Senthilkumar K., Vandamme E., Akakpo C., Allarangaye M.D., Baggie I., Bakare S.O., Bam R.K. (2019). Status quo of chemical weed control in rice in sub-Saharan Africa. Food Secur..

[14] Rohlman D.S., Davis J.W., Ismail A., Rasoul G.M.A., Hendy O., Olson J.R., Bonner M.R. (2020). Risk perception and behavior in Egyptian adolescent pesticide applicators: An intervention study. BMC Public Health.

[15] Röösli M., Fuhrimann S., Atuhaire A., Rother H.-A., Dabrowski J., Eskenazi B., Jørs E., Jepson P.C., London L., Naidoo S. (2022). Interventions to reduce pesticide exposure from the agricultural sector in Africa: A workshop report. Int. J. Environ. Res. Public Health.

[16] Fuhrimann S., Wan C., Blouzard E., Veludo A., Holtman Z., Chetty-Mhlanga S., Dalvie M.A., Atuhaire A., Kromhout H., Röösli M. (2021). Pesticide research on environmental and human exposure and risks in sub-Saharan Africa: A systematic literature review. Int. J. Environ. Res. Public Health.

[17] Ndayambaje B., Amuguni H., Coffin-Schmitt J., Sibo N., Ntawubizi M., VanWormer E. (2019). Pesticide application practices and knowledge among small-scale local rice growers and communities in Rwanda: A cross-sectional study. Int. J. Environ. Res. Public Health.

[18] Umubyeyi S., Rukazambuga N. (2016). Small scale farmers’ knowledge on grain losses from bean bruchid, pesticides safe use and implication on food security and safety in Huye District, Rwanda. Rwanda J..

[19] Antoine N.M., Emmanuel K., Claudine N., Koca I. (2022). Assessment of the pesticides utilization and the pesticide residues presence in fresh and tomato products for the tomato supply chain in Rwanda. Food Nutr. Sci..

[20] Tambo J.A., Kansiime M.K., Mugambi I., Rwomushana I., Kenis M., Day R.K., Lamontagne-Godwin J. (2020). Understanding smallholders’ responses to fall armyworm (*Spodoptera frugiperda*) invasion: Evidence from five African countries. Sci. Total Environ..

[21] Tambo J.A., Romney D., Mugambi I., Mbugua F., Bundi M., Uzayisenga B., Matimelo M., Ndhlovu M. (2021). Can plant clinics enhance judicious use of pesticides? Evidence from Rwanda and Zambia. Food Policy.

[22] Tambo J.A., Uzayisenga B., Mugambi I., Onyango D.O., Romney D. (2023). Sustainable management of fall armyworm in smallholder farming: The role of a multi-channel information campaign in Rwanda. Food Energy Secur..

[23] Kwizera E., Rumbeiha W.K., Nishimwe K., Nziza J. (2024). A survey to document toxic hazards in the zone surrounding volcanoes national park, a habitat for mountain gorillas, an endangered wildlife species in Rwanda. Front. Vet. Sci..

[24] Fasina F.O., Fasanmi O.G., Makonnen Y.J., Bebay C., Bett B., Roesel K. (2021). The One Health landscape in Sub-Saharan African countries. One Health.

[25] Pitt S.J., Gunn A. (2024). The One Health Concept. Br. J. BioMed Sci..

[26] Rwanda Agriculture and Animal Resources Development Board (RAB) (2025). Rwanda Agriculture and Animal Resources Development Board.

[27] National Agricultural Export Development Board (NAEB) (2025). National Agricultural Export Development Board.

[28] Republic of Rwanda The Government of Rwanda 2025. https://www.gov.rw/overview.

[29] Hegarty-Craver M., Polly J., O’neil M., Ujeneza N., Rineer J., Beach R.H., Lapidus D., Temple D.S. (2020). Remote crop mapping at scale: Using satellite imagery and UAV-acquired data as ground truth. Remote Sens..

[30] National Institute of Statistics of Rwanda (NISR) (2024). Seasonal Agricultural Survey, Season A 2024 Report.

[31] Hennink M.M., Kaiser B.N., Weber M.B. (2019). What influences saturation? Estimating sample sizes in focus group research. Qual. Health Res..

[32] Morgan D.L. (1996). Focus groups. Annu. Rev. Sociol..

[33] Morgan D.L. (1997). Focus Groups as Qualitative Research.

[34] Caillaud S., Nikos K., Doumergue M., Flick U. (2022). Designing focus groups. The Sage Handbook of Qualitative Research Design.

[35] Tang K.C., Davis A. (1995). Critical factors in the determination of focus group size. Fam. Pract..

[36] Parker C., Scott S., Geddes A. (2019). Snowball sampling. SAGE Res. Methods Found..

[37] Chand S.P. (2025). Methods of data collection in qualitative research: Interviews, focus groups, observations, and document analysis. Adv. Educ. Res. Eval..

[38] Braun V., Clarke V. (2006). Using thematic analysis in psychology. Qual. Res. Psychol..

[39] Maguire M., Delahunt B. (2017). Doing a thematic analysis: A practical, step-by-step guide for learning and teaching scholars. All. Irel. J. High. Educ..

[40] Nziza A.-R. (2020). IPEN Toxics-Free SDGs Campaign Highly Hazardous Pesticides (HHPs) Rwanda Situation Report.

[41] World Health Organization (2020). WHO Recommended Classification of Pesticides by Hazard and Guidelines to Classification, 2019 Edition.

[42] Law on Governing of Agrochemicals, Law N° 30/2012 of 1 August 2012, Official Gazette of the Republic of Rwanda (2012). https://rwandalii.org/akn/rw/act/law/2012/30/eng@2012-09-10.

[43] World Bank (2025). Population density (people per sq. km of land area). Food and Agriculture Organization and World Bank Population Estimates.

[44] National Institute of Statistics of Rwanda (NISR) (2023). Fifth Rwanda Population and Housing Census, Main Indicators Report.

[45] Austin K.G., Beach R.H., Lapidus D., Salem M.E., Taylor N.J., Knudsen M., Ujeneza N. (2020). Impacts of climate change on the potential productivity of eleven staple crops in Rwanda. Sustainability.

[46] National Institute of Statistics of Rwanda (NISR) (2013). Seasonal Agricultural Survey 2013, Version 2.

[47] National Institute of Statistics of Rwanda (NISR) (2024). Seasonal Agricultural Survey, Season B Annual Report, December 2024.

[48] Rwema M., Safari B., Laine M., Sylla M.B., Roininen L. (2025). Trends and Variability of Temperatures in the Eastern Province of Rwanda. Int. J. Climatol..

[49] Timilsena B.P., Niassy S., Kimathi E., Abdel-Rahman E.M., Seidl-Adams I., Wamalwa M., Tonnang H.E.Z., Ekesi S., Hughes D.P., Rajotte E.G. (2022). Potential distribution of fall armyworm in Africa and beyond, considering climate change and irrigation patterns. Sci. Rep..

[50] Goergen G., Kumar P.L., Sankung S.B., Togola A., Tamò M. (2016). First report of outbreaks of the fall armyworm Spodoptera frugiperda (JE Smith) (Lepidoptera, Noctuidae), a new alien invasive pest in West and Central Africa. PLoS ONE.

[51] Ministry of Agriculture and Animal Resources (MINAGRI) (2017). Government Intensifies Efforts to Contain Fall Armyworms [Press Release]. https://www.minagri.gov.rw/updates/news-details/government-intensifies-efforts-to-contain-fall-armyworms.

[52] Uzayisenga B., Bizimana J.P., Dusengemungu L., Karangwa P., Rukundo P., Niassy S., Ekesi S., Migiro L., Otieno W. (2020). Farmers’ perceptions and preferences on pesticide use in the management of fall armyworm in Rwanda. Sustainable Management of Invasive Pests in Africa.

[53] Rukundo P., Karangwa P., Uzayisenga B., Ingabire J.P., Waweru B.W., Kajuga J., Bizimana J.P., Niassy S., Ekesi S., Migiro L., Otieno W. (2020). Outbreak of fall armyworm (*Spodoptera frugiperda*) and its impact in Rwanda agriculture production. Sustainable Management of Invasive Pests in Africa.

[54] Jepson P.C., Murray K., Bach O., Bonilla M.A., Neumeister L. (2020). Selection of pesticides to reduce human and environmental health risks: A global guideline and minimum pesticides list. Lancet Planet. Health.

[55] Chandrasena D.I., Signorini A.M., Abratti G., Storer N.P., Olaciregui M.L., Alves A.P., Pilcher C.D. (2018). Characterization of field-evolved resistance to Bacillus thuringiensis-derived Cry1F δ-endotoxin in Spodoptera frugiperda populations from Argentina. Pest Manag. Sci..

[56] Gutiérrez-Moreno R., Mota-Sanchez D., Blanco C.A., Whalon M.E., Terán-Santofimio H., Rodriguez-Maciel J.C., DiFonzo C. (2019). Field-evolved resistance of the fall armyworm (Lepidoptera: Noctuidae) to synthetic insecticides in Puerto Rico and Mexico. J. Econ. Entomol..

[57] Zhang L., Liu B., Zheng W., Liu C., Zhang D., Zhao S., Li Z., Xu P., Wilson K., Withers A. (2020). Genetic structure and insecticide resistance characteristics of fall armyworm populations invading China. Mol. Ecol. Resour..

[58] Carvalho R.A., Omoto C., Field L.M., Williamson M.S., Bass C. (2013). Investigating the molecular mechanisms of organophosphate and pyrethroid resistance in the fall armyworm Spodoptera frugiperda. PLoS ONE.

[59] Aminiahidashti H., Jamali S.R., Gorji A.M.H. (2014). Conservative care in successful treatment of abamectin poisoning. Toxicol. Int..

[60] Guo L., Zhou Z., Dai P., Zhang T., Genjiafu A., Jian T., Wen Z., Zhao L., Li Q., Jian X. (2023). Case report: Occupational acute poisoning caused by the accidental release of lambda-cyhalothrin. Front. Environ. Health.

[61] Kushwaha M., Verma S., Chatterjee S. (2016). Profenofos, an acetylcholinesterase-inhibiting organophosphorus pesticide: A short review of its usage, toxicity, and biodegradation. J. Environ. Qual..

[62] Priyanka A., Raj A., Madan P., Rani S. (2020). Adverse effects of cypermethrin: A review. Int. J. Sci. Technol. Res..

[63] Elsharkawy E.E., Abd El-Nasser M., Bakheet A.A. (2019). Mancozeb impaired male fertility in rabbits with trials of glutathione detoxification. Regul. Toxicol. Pharmacol..

[64] Mohammadi-Sardoo M., Mandegary A., Nabiuni M., Nematollahi-Mahani S.-N., Amirheidari B. (2018). Mancozeb induces testicular dysfunction through oxidative stress and apoptosis: Protective role of N-acetylcysteine antioxidant. Toxicol. Ind. Health.

[65] European Union (EU) (2020). Mancozeb Non-Renewal and MRL Review.

[66] Rwanda Environment Management Authority (REMA) (2011). The National Integrated Pest Management (IPM) Framework for Rwanda, Final Draft Report.

[67] Sanon A., De Bauw P., Lehmann E., Muriuki L., Compaoré I., Sanou M.R., Lopes T. (2025). Development of viable Integrated Pest Management (IPM) strategies to control fall armyworm (*Spodoptera frugiperda*) on maize. Crop Prot..

[68] Atinkut Asmare B., Freyer B., Bingen J. (2022). Women in agriculture: Pathways of pesticide exposure, potential health risks and vulnerability in sub-Saharan Africa. Environ. Sci. Eur..

[69] Alshalati L.M.J. (2021). Limited knowledge and unsafe practices in usage of pesticides and the associated toxicity symptoms among farmers in Tullo and Finchawa rural kebeles, Hawassa City, Sidama Regional State, Southern Ethiopia. Emerging Contaminants.

[70] Zseleczky L., Christie M.E., Haleegoah J. (2014). Embodied livelihoods and tomato farmers’ gendered experience of pesticides in Tuobodom, Ghana. Gend. Technol. Dev..

[71] Tsimbiri P.F., Moturi W.N., Sawe J., Henley P., Bend J.R. (2015). Health impact of pesticides on residents and horticultural workers in the Lake Naivasha Region, Kenya. Occupat Dis. Environ. Med..

[72] Christie M.E., Van Houweling E., Zseleczky L. (2015). Mapping gendered pest management knowledge, practices, and pesticide exposure pathways in Ghana and Mali. Agric. Hum. Values.

[73] Sankoh A.I., Whittle R., Semple K.T., Jones K.C., Sweetman A.J. (2016). An assessment of the impacts of pesticide use on the environment and health of rice farmers in Sierra Leone. Environ. Int..

[74] Mwabulambo S.G., Mrema E.J., Ngowi A.V., Mamuya S. (2018). Health symptoms associated with pesticides exposure among flower and onion pesticide applicators in Arusha region. Ann. Glob. Health.

[75] Ochago R. (2018). Gender and pest management: Constraints to integrated pest management uptake among smallholder coffee farmers in Uganda. Cogent Food Agric..

[76] World Economic Forum (2025). Global Gender Gap Report 2025.

[77] Gahigi M. (2025). Rwanda Bees Being Wiped out by Pesticides.

[78] Akça R., Saruhan I. (2022). The effects of some insecticides on honeybees (*Apis mellifera*). Isr. J. Ecol. Evol..

[79] Belsky J., Biddinger D.J., Seiter N., Joshi N.K. (2022). Various routes of formulated insecticide mixture whole-body acute contact toxicity to honey bees (*Apis mellifera*). Environ. Chall..

[80] Gordon I. (2018). Livestock production increasingly influences wildlife across the globe. Animal.

[81] Reif J.S. (2011). Animal sentinels for environmental and public health. Public Health Rep..

[82] Rial-Berriel C., Acosta-Dacal A., Pérez M.Á.C., Suárez-Pérez A., Melián A.M., Zumbado M., Hernández L.A.H., Ruiz-Suárez N., Hernández Á.R., Boada L.D. (2021). Intensive livestock farming as a major determinant of the exposure to anticoagulant rodenticides in raptors of the Canary Islands (Spain). Sci. Total Environ..

[83] Berny P. (2007). Pesticides and the intoxication of wild animals. J. Vet. Pharmacol. Ther..

[84] Maurer B.A., Holt R.D. (1996). Effects of chronic pesticide stress on wildlife populations in complex landscapes: Processes at multiple scales. Environ. Toxicol. Chem..

[85] Sánchez C.A., Altizer S., Hall R.J. (2020). Landscape-level toxicant exposure mediates infection impacts on wildlife populations. Biol. Lett..

