# Supplementary material for: Pesticide Use and Safety Behaviors Among Farmers in Rwanda’s Eastern Province: Perspectives from Sector Officers on Drivers, Factors, and Gender Differences
Source: Int J Environ Res Public Health. 2026 Jun 8;23(6):771. doi: 10.3390/ijerph23060771 (PMC13299060; doi:10.3390/ijerph23060771)
Supplement: Supplementary file 1 [file ijerph-23-00771-s001.zip › S1_Protocol.pdf]

FOCUS GROUP PROTOCOL
Project: Pesticide Risks and Female Health in Rwanda

Setup
Participants: Around 5 individuals per group
Team members: one moderator and 2-4 assistant moderators for notetaking, audio recording, and logistics
Setting: large and comfortable space with privacy and circle seating
Refreshments: Water, soft drinks, light snacks
Duration: 1-2 hours
Documentation: typed notes, digital voice records, consent forms

Procedure
We conducted one FGD in each of the five selected districts during the period of December 16-20, 2024. Each FGD was held in the conference room of a hotel in the district, which allowed privacy and accessibility for the participants. We conducted each FGD with one moderator (Rwibutso), two notetakers (Irimaso and Nyirahabimana), and two observers in support roles (Curl and Sholts). Before each discussion began, each participant received a written overview of the project and details about participation in a Consent Form, which Rwibutso also explained verbally in Kinyarwanda (see "Script Outline" below). This pre-discussion introduction included several key points:

1. Our team devised a series of measures to safeguard the confidentiality of participants and protect any data that we collected from them. The signed Consent Forms would be stored in a secure, locked file cabinet that would only be accessible to one team member (Sholts) at her institution. The discussion would not include any names, and the audio recording of the discussion would be stored on a pin code-protected device in the possession of a team member at all times. After the English-translated transcription of the discussion was completed by our team, the audio recording would be destroyed. The transcription would not include or be associated with any names, and all data would be presented/published without identifying information and summarized or combined with data from all of the study's participants. The Consent Forms and data would be kept for three years after the end of the study and then destroyed.
2. Each participant would receive 40,000 RWF as compensation for their time and transportation expenses to/from the venue for the discussion.
3. Participation was voluntary. We did not anticipate that any questions would cause personal discomfort or risk harm to any participants, but clarified that participants were free to decline to answer any questions or to stop their participation in the discussion at any time and still receive their full compensation. However, we explained that it would not be possible to remove a participant's responses from our data because their comments would be anonymous and so we would not know which ones were theirs.
4. All participants received local contact information for one of us (Irimaso) to address any questions or concerns about their participation in the study after leaving the venue. In case they had any questions about their rights as a research participant, we also provided

them with contact information for the person responsible for the protection of volunteers in research projects at the Directorate of Research and Innovation in the College of Agriculture, Animal Sciences and Veterinary Medicine at the University of Rwanda (which granted ethical clearance for the study).

Script Outline

- **Introduction**

Good afternoon and welcome to this focus group session.

Please note that we are using a voice recording device, and we're hitting record now.

At this point, the audiorecorder will visibly press the 'play' button on a voice recording device that will be prominently positioned in close proximity to the participants.

We are recording the session because we don't want to miss any of your comments. People often say very helpful things in these discussions, and we can't type fast enough to write them all down. We are not using any names in this discussion, and we won't use any names or other identifiable information in our data analysis, report, or publications. You should therefore feel complete confidentiality in the information and opinions that you share with us.

Thank you for taking the time to speak with us today. My name is [NAME], and I will serve as the moderator of our discussion. Assisting me are my colleagues, [NAMES], who are taking notes, audio recording, and providing logistical support.

Our topic for this conversation is pesticide use in agriculture in Rwanda. We are interested in how farm workers acquire and use pesticides for their various tasks and other ways that they may be exposed to them.

Our goal is to identify potential health risks associated with different behaviors and activities involving pesticides, so that we can make recommendations for safer practices. We are especially interested in the potential health risks for women and animals, because they have not been the focus of a lot of research to date. We also hope to gather some information that will help us to design future focus groups involving the farm workers themselves.

Your responses will be combined with other focus groups for analysis, and we plan to summarize our findings in a report and scientific articles that will be shared with you when they are complete.

- **Guidelines**

You have been invited to participate in this study because of your expertise in this topic as an agricultural officer in this region of Rwanda. We will be asking you questions that relate to your own observations and knowledge, so there are no wrong answers. Please feel free to share your point of view, even if it differs from what others have said. Of course, you are free to not respond to a question if you prefer. You are welcome to speak to each other, but only one person should speak at a time.

We will be covering a number of different subtopics in our conversation, and we will try to spend 10 to 20 minutes on each one. Our timekeeper will help us stay on track, and I will let you know when we are shifting from one subtopic to another.

If all of that is clear, then let's begin.

- **Questions**

I would like to start by learning more about your thoughts on pesticide use in general.

a. **General use of pesticides in agriculture**

- How would you characterize agriculture in this region compared to other parts of Rwanda?
    - How many people are involved in agricultural work?
    - What kind of produce is grown?
- Are you aware of any changes in pesticide use in Rwanda agriculture in recent decades?
    - How does pesticide use today differ from the past?
    - Why do you think there have been changes?
- Which pesticides are most heavily used by farm workers today - insecticides, herbicides, or fungicides?
- How do farm workers acquire pesticides?
    - Where do they buy them?
    - How do they decide which products and quantities to buy?
    - Are pesticides ever sold or stored in unlabeled containers?
    - When pesticide containers are labeled, are most workers able to read the information provided on the label?
- How much information do farm workers receive about health risks and safe practices involving pesticides?
    - Where does the information come from?
    - Are there barriers to receiving or understanding the information?
- In general, what kind of personal protective equipment (PPE) do farm workers wear during pesticide use?
    - Do they receive training for the PPE?
    - Are there barriers to acquiring or using it?

b. **Specific uses of pesticides by farm workers**

Now I would like to focus our conversation on specific uses of pesticides by farm workers.

- What are the most common activities that involve pesticides?
  - How are pesticides typically applied?
  - How often are pesticides applied to crops?
  - Does the frequency vary between types of crops?
- How much or how often is PPE worn during these different activities?

c. **Gender difference in uses of pesticides by farm workers**

Now let's focus on female farm workers in particular.

- About what percentage of farm workers in this region are women?
- Is there a division of labor between men and women generally?
  - What are the most common tasks or duties for female workers?
  - Do both men and women mix and apply pesticides, or are there differences in these tasks by gender?
- Do you think there are any differences in how men and women agricultural workers perceive pesticide risks and safety behaviors?
- Are there women less likely to seek information about the safe use of pesticides? Why do you think this is?
- Are women less likely to wear PPE? Why do you think this is?

At this point, I would like to hear a little bit about how animals may be exposed to or affected by pesticide use by farm workers.

d. **Animal involvement in uses of pesticides by farm workers**

- Are there livestock that are kept in the same spaces where produce is grown?
  - If so, what kind and how many?
- Are there any other domestic animals (dogs or cats) that roam or share the same spaces where produce is grown?
- Are animals ever exposed directly to pesticides by farm workers, either intentionally or not?

Now that we've discussed quite a bit about pesticides, let's turn our conversation to the health implications.

e. **Potential health effects of pesticide use or exposure**

- Do you have any concerns about potential human health effects from pesticide use among agricultural workers?
- Have you heard any agricultural workers reporting adverse health effects that they think are due to pesticides?

Thank you for all the information that you've shared about pesticide use by farm workers. Now I would like to invite you to share your thoughts and opinions about what kinds of things might help the situation.

f. Priority concerns involving pesticides in agriculture

- What kind of changes in pesticide use would you like to see in this region?
- What kind of information or initiative would help make this happen?

In our final section, I would like to ask you for your advice on how we can recruit participants and conduct more focus groups of farm workers.

g. Advice for future research

- Do you think that farm workers would be interested in participating in these focus groups?
- Are there any challenges that we should consider for male or female participants specifically?

Conclusion

To close, is there anything else about pesticide use in Rwandan agriculture that you would like to talk about?

Thank you for sharing your thoughts with us today. You have each been extremely valuable in helping us understand more about pesticide use among farm workers. Your responses will inform our study about potential health risks associated with different behaviors and activities involving pesticides, so that we can make recommendations for safer practices.

Please see my colleague [NAME] to receive your compensation and travel reimbursement before you leave. Have a nice day. Goodbye!
