# Supplementary material for: Pesticide Use and Safety Behaviors Among Farmers in Rwanda’s Eastern Province: Perspectives from Sector Officers on Drivers, Factors, and Gender Differences
Source: Int J Environ Res Public Health. 2026 Jun 8;23(6):771. doi: 10.3390/ijerph23060771 (PMC13299060; doi:10.3390/ijerph23060771)
Supplement: Supplementary file 1 [file ijerph-23-00771-s001.zip › S1_Table.pdf]

Category	Mechanism of Action	Chemical Class	Active Ingredient	Trade Name	Regions mentioned
Insecticides	Neurotoxins	Pyrethroids	Cypermethrin (including alpha- and beta-)	Alpha +<br>Simba+	Rwamagana, Nyagatare, Kayonza, Kirehe, Ngoma
			Lambda-cyhalothrin	Jackmax<br>Lambdex<br>Ramda<br>Lambda +	Rwamagana, Nyagatare, Kayonza, Kirehe, Ngoma
		Avermectins	Abamectin	Dudu Aba+<br>Supa Dudu<br>Dudu<br>Dudu +	Rwamagana, Nyagatare, Kayonza, Kirehe, Ngoma
			Emamectin benzoate	Super Coma<br>L-Bencol	Kirehe
		Organophosphates	Profenfos		Nyagatere
		Organochlorines	Endosulfan	Thiodan	Kirehe
		Neonicotinoids	Thiamethoxam<br>Imidacloprid<br>Dinotefuran	 Vital<br>Safari	Nyagatere<br>Nyagatere<br>Rwamagana
		Organophosphate & Pyrethroid	Mix of Cypermethrin & Profenfos	Cypro 44EC<br>Profex<br>Profex Super<br>Rocket<br>Roket	Rwamagana, Nyagatare, Kayonza, Kirehe, Ngoma
	Growth regulators	Chitin synthesis inhibitor	Buprofezin		Nyagatere
	Respiratory system disruptor	Phosphine gas	Aluminum phosphide	Quickphos	Kirehe
	Other (Neurotoxin + energy metabolism disregulator)	Pyrazole & Pyrethroid	Mix of Tofenpyrad & Bifenthrin	Raavan	Kayonza, Ngoma
Insecticides	Neurotoxins	Pyrethroids	Deltamethrin	Decatrix	Nyagatere

(Animal use, mites & ticks)			Cypermethrin (including alpha- and beta-)	Ashimetrin	Kayonza
		Amidines	Amitraz	Norotraz	Ngoma
Fungicides	Enzyme inhibitor (sulfhydral groups)	Dithiocarbamate	Mancozeb	Mancobex<br>Dithane<br>Indothane<br>Agrothane P+	Rwamagana, Nyagatare, Kayonza, Kirehe, Ngoma
	Growth and metabolism interference	"organic"	Copper oxychloride		Rwamagana, Nyagatere
			Sulfur	Sulfur 80 WDG	Nyagatere
	Fungal RNA synthesis disruptor	Phenylamine	Metalaxyl		Nyagatere
	Protein inhibitor (tubulin)	Benimidazole	Carbendazim		Nyagatere
			Tricyclazole		Nyagatere
	Mixtures	Dithiocarbamate & Phenylamine	Mancozeb & Metalaxyl	Radomol	Rwamagana, Nyagatere
				Ridomil	Rwamagana, Nyagatare, Kayonza, Kirehe, Ngoma
Herbicides	Cell division and growth disruptor	Hydrazine	Hydrazine		Nyagatere, Kyonza
		Pheyoxyacetic acid	2.4-D	Tzone	Nyagatere

S1 Table. A complete list of active ingredients mentioned in focus groups and observed in Agro&Vet shops in all five districts. Six compounds were mentioned that are unknown and that we have not been able to associate with any known pesticide: achimetrine, copi, tiamidor, decamix, parmapy plus, radomouri, and pesto-agro.
